# Growth Hormone and Endometrial Receptivity

**DOI:** 10.3389/fendo.2019.00653

**Published:** 2019-09-24

**Authors:** Signe Altmäe, Lusine Aghajanova

**Affiliations:** ^1^Department of Biochemistry and Molecular Biology, Faculty of Sciences, University of Granada, Granada, Spain; ^2^Competence Centre on Health Technologies, Tartu, Estonia; ^3^Instituto de Investigación Biosanitaria ibs.GRANADA, Granada, Spain; ^4^Division of Reproductive Endocrinology and Infertility, Department of Obstetrics and Gynecology, Stanford School of Medicine, Sunnyvale, CA, United States

**Keywords:** endometrium, endometrial receptivity, growth hormone, infertility, *in vitro* fertilization, transcriptome

## Abstract

Administration of growth hormone (GH) during ovarian stimulation has shown beneficial effects on *in vitro* fertilization (IVF) outcomes. It is generally believed that this improvement is due to the stimulating effect of GH on oocyte quality. However, studies are emerging that show possible positive effect of GH administration on endometrial receptivity, thus suggesting an additional potential benefit at the level of the uterus, especially among women with recurrent implantation failure, thin endometrium, and older normal responders. This review summarizes recent data on GH co-treatment effects on endometrium and endometrial receptivity among infertile women undergoing IVF, and proposes possible mechanisms of GH actions in the endometrium.

## Introduction

Receptive endometrium is an absolute prerequisite for a successful embryo implantation, being defined by a limited time-frame when the endometrium is favorable for embryo adhesion and the subsequent attachment and invasion processes (1).

Endometrial receptivity is a complex process that is orchestrated by the synergistic actions of main reproductive hormones estrogen and progesterone, as well as plead of other endocrine, paracrine and autocrine factors (2, 3). Impaired endometrial receptivity is thought to be one of the major reasons for embryo implantation failure (4). In assisted reproductive technologies (ART), where the good quality embryos are transferred as a standard of care, implantation failure remains an unsolved obstacle (5, 6). Regardless of the advances in assisted reproduction, particularly regarding the more effective means of embryo selection and cryopreservation, many patients repeatedly fail the treatment procedure. What we are facing today is that implantation failure in ART is common, and we lack the evidence-based therapeutic solutions for treating it. As a result, clinicians often feel obliged to offer treatments that are largely empirical, based on some biologic rationale but with little clinical evidence to support their use (7, 8). The treatment failure is equally frustrating to both patients and their providers, which even more emphasizes the urgent need for novel effective treatment to prevent yet another failure.

The role of growth hormone (GH) in female reproduction has gained renewed interest and has become a heated topic over the last decade. The local GH production in the reproductive tissues themselves exert an important autocrine/intracrine effects on those tissues, in addition to the pituitary production of GH (9). Moreover, local insulin growth factor 1 (IGF-1) production (known downstream mediator of GH) has been shown to be controlled by gonadotropins and estradiol as well (10). Evidence emerging from clinical practice suggests that GH administration during ovarian stimulation may improve oocyte quality [higher number of oocytes collected, higher fertilization rate, and higher number of embryos reaching the transfer stage (11–15)], increase pregnancy rate (16–24), implantation rate (16, 20–23, 25, 26), and live birth rate (12, 16, 19, 20, 23, 25, 27). The accumulating beneficial effects of GH on assisted reproduction outcomes do not exclude the possibility that this effect is due, at least in part, to an action of GH on endometrial receptivity.

## Growth Hormone in the Endometrium

GH is a peptide hormone secreted by the anterior pituitary gland, having important role in cell growth and metabolism throughout the body. GH together with its receptor, GHR, and related growth factors including IGF-1, is expressed in the endometrium of rats and human (28–31). The study by Sbracia et al. obtained biopsies from women in proliferative and secretory phases, as well as first trimester decidua (from elective pregnancy terminations) (28). They showed that there was no GH expression in proliferative glandular epithelium, but GH immunoreactivity appeared in the mid-luteal secretory phase (no subdivision within secretory phase was done) and increased in the decidua from the first trimester abortions, with similar expression in the decidual samples from the term pregnancies, suggesting a role in embryo implantation process. Interestingly, no stromal expression of GH was observed in any sample (28). Moreover, the authors analyzed GH expression in the endometrium from women with “luteal phase defect,” defined by low progesterone levels <8 ng/mL and delayed endometrial maturation, and saw significantly lower expression of GH (28). This data suggested close relationship between GH expression in endometrium and progesterone level/function. Further, a recent study on human endometrial cell line indicated that GH may act in a direct or IGF-1-mediated manner on human endometrial cells to promote proliferation and vascularization and up-regulation of receptivity-related genes such as vascular endothelial growth factor (VEGF) and integrin beta 3 (ITGB3) (21). VEGF is an important player in angiogenesis (32), and it has been shown to act in an autocrine manner on endometrial epithelial cell adhesion as a key regulator in the implantation process (33). ITGB3 is a well-known biomarker of receptivity (34), and down-regulation of this biomarker (phenomenon detected in women with unexplained infertility, endometriosis, and luteal phase deficiency) has been related to lower pregnancy rates (35, 36).

**Figure 1:**
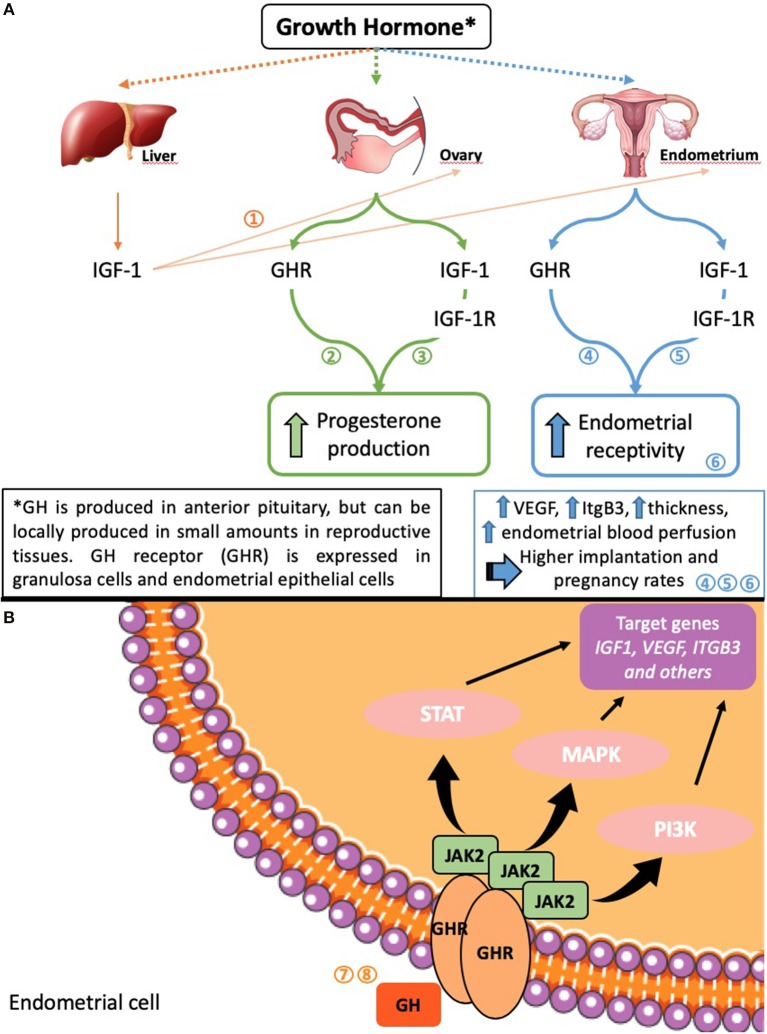
Possible mechanisms of GH effects on ovarian and endometrial function **(A)** and on endometrial cells **(B)**. Numbers in the figure indicate studies where the information is presented in detail: 1 (39); 2 (40, 41); 3 (42); 4 (23); 5 (21); 6 (43); 7 (44); 8 (45).

Apart from the effects of circulating GH and locally produced GH on endometrium, there is a proposed indirect effect of ovarian GH on endometrial function, namely its involvement in the function and maintenance of the corpus luteum (37, 38). While the majority of the data come from various animal models, they are nevertheless significant. Luteal function and its maintenance are vital for the establishment of pregnancy and its viability due to the production of progesterone by the corpus luteum—the main “keeper” of the normal early pregnancy. Hence, the stimulatory effect of GH on ovarian steroidogenic cell function may play a major role in endometrial function and dysfunction via its effect on ovary (see Figure 1 for the proposed mechanisms of GH action on endometrium).

## Clinical Use of GH and Effect on the Endometrium

Initial reports on the use of GH in clinical practice come from cases of hypogonadotropic hypogonadism or panhypopituitarism (46). Subsequently, the use of GH has been expanded on different patient population, such as women with poor ovarian reserve, poor responders, or with poor oocyte quality due to advanced maternal age (25, 47, 48). In general, GH administration in the infertility clinic setting has focused on GH effects on oocyte, and the effect on endometrium has been largely overlooked.

Subsequently, the attention has been turned onto the endometrium, and interesting observations have been made suggesting positive effect of growth hormone treatment on endometrial thickness and implantation potential (see Table 1 for the studies). A case report of a patient with panhypopituitarism demonstrated improved endometrial thickness and successful implantation and pregnancy after adding growth hormone to the treatment protocol following multiple failed *in vitro* fertilization (IVF)/embryo transfer cycles (55). Alternatively, a study on 20 patients with documented GH deficiency reported improved embryo quality, but no improvement in endometrial thickness, when supplemented with GH in IVF cycle (15) (Table 1). Below we will discuss the available literature on the use of GH in various clinical IVF settings.

**Table 1:** Studies assessing the effect of growth hormone (GH) co-treatment in *in vitro* fertilization (fresh treatment cycles and frozen embryo transfer cycles) on endometrium.

**Study**	**RCT**	**Study group; Ethnicity**	**GH/control (mean age)**	**Inclusion criteria**	**Exclusion criteria**	**Intervention**	**Primary outcome**	**Effect on endometrial thickness (mm)**		
								**GH**	**Control**	***p*-value**
**FRESH EMBRYO TRANSFER CYCLE**										
Rajesh et al. (15)	No	Infertile women with GH deficiency; Chinese	20/20^*^ (32.9 y) ^*^same women cycle before without GH served as controls	GH deficiency based on clonidine test; previous IVF cycle without GH; became pregnant with GH treated cycle	Panhypopituitarism; GH deficient patients with previous cycle treated at other hospital	12 IU GH every 3rd day, starting from GnRH stimulation day until hCG administration	Improved embryo quality; higher fertilization rate at ICSI	11.4 ± 1.9	10.3 ± 1.5	0.108
Eftekhar et al. (49)	Yes	Poor responders; Iranian	40/42 (36.0 ± 4.6 y/36.2 ± 3.7 y)	previous failed IVF-ET cycles with ≤ 3 oocytes, and ≤ 3 embryos obtained; and/or E2 levels ≤ 500 pg/mL on hCG day	BMI ≥30, FSH >15 IU/L, endocrine or metabolic disorders, and PCOS, severe endometriosis and azoospermia	GnRH antagonist protocol; +treatment group 4 IU/d GH from day 21 from previous cycle until hCG triggering	Higher number of retrieved oocytes and obtained embryos, while no effect on implantation and pregnancy rates	8.5 ± 1.0	8.1 ± 0.9	0.158^a^
Bayoumi et al. (50)	Yes	Poor responders; Egyptian	72/73 (34.9 ± 4.9 y/34.8 ± 5.6 y)	ESHRE consensus criteria 2011 for poor responders	FSH >20 IU/l; previous ovarian surgery; infertility other than poor ovarian response; endocrine disorder; male factor infertility	GnRH agonist (microflare) protocol; +treatment group 7.5 IU/d GH from day 6 of hMG stimulation until day of hCG triggering	Higher number of mature oocytes and embryos obtained, while no effect on implantation and pregnancy rates	11.9 ± 1.6	11.7 ± 1.7	0.590^a^
Dakhly et al. (51)	Yes	Poor responders; Egyptian	74/74/68/71^*^ (36.4 ± 5.8 y/38.1 ± 5.0 y/36.8 ± 6.3 y/36.4 ± 5.8 y) ^*^Comparison of 4 different GH protocols, no control group	ESHRE consensus criteria 2011 for poor responders	>45 y; FSH >20 IU/l; previous ovarian surgery; other causes of infertility (other than poor responder); male factor of infertility	Gr1: GnRH long protocol; Gr2: GnRH short protocol; Gr3: GnRH antagonist protocol; Gr4: GnRH miniflare protocol. In all groups 7.5 IU/d GH from day 6 of hMG stimulation until day of hCG triggering	The long/GH (Gr1) protocol was superior regarding the number of oocytes retrieved and fertilized. No significant differences in pregnancy rates	11.5 ± 1.6 (Gr1); 11.4 ± 1.6 (Gr2) **12.1 ± 1.4 (Gr3); 11.1 ± 1.8 (Gr4)**	NA	**0.003^a^** (Gr3 vs. Gr4)
Bassiouny et al. (13)	Yes	Poor responders; Egyptian	68/73 (35.8 ± 5.6 y/35.5 ± 6.0 y)	ESHRE consensus criteria 2011 for poor responders	FSH >20 IU/l; previous ovarian surgery; infertility other than poor ovarian response	GnRH antagonist protocol; +treatment group 7.5 IU/d GH from day 6 of hMG stimulation until day of hCG triggering	Higher number of mature oocytes and embryos obtained, while no effect on pregnancy rates	12.1 ± 1.3	11.6 ± 1.6	**0.029^a^**
Du et al. (16)	No	Normal responders; Chinese	556/558 (32.8 ± 4.3 y/31.6 ± 4.4 y) (^*^older women ≥**35 y: 278/265**; ^**^younger women <35 y: 278/293)	20-45 y; fallopian tube malfunction or male sterility; normal hormone levels; normal uterine cavity; regular menstrual cycles, BMI <25	Recurrent spontaneous abortion; severe pelvic adhesions or hydrosalpinx; cerebrovascular, liver or kidney disease; endocrine diseases; PCOS; endometriosis; uterine leiomyoma; adenomyosis	Long GnRH agonist protocol; +treatment group 4.5 IU/d GH for 5 days starting from day of FSH administration	Higher implantation and clinical pregnancy rates	12.2 ± 4.7 ^*^12.0 ± 2.2 ^**^12.5 ± 7.0	11.8 ± 4.8 ^*^11.6 ± 2.5 ^**^12.0 ± 6.8	0.18^b^ ^*^0.038^b^ ^**^0.50^b^
Choe et al. (52)	Yes	Infertile women with diminished ovarian reserve; Korean	62/65 (39.8 ± 3.6 y/39.4 ± 4.1 y)	≥40 y or any other factor for poor ovarian response; ≤ 3 oocytes with conventional stimulation protocol; antral follicle count <5–7 or AMH <0.5–1.1 ng/ml; normal uterus; regular menstrual cycle	Genetic cause for infertility; BMI >30; abnormal uterine bleeding; ovarian tumor; breast cancer; hydrosalpinx; contraindication for GH treatment	GnRH antagonist protocol; +treatment group sustained-release GH (20 mg) 3 × before and during COS (mid-luteal, late luteal, cycle day 2)	Higher number of mature oocytes obtained, while no effect on pregnancy rates	8.8 ± 2.2	9.1 ± 1.9	0.24^a^
Dakhly et al. (53)	Yes	Poor responders; Egyptian	120/120 (36.4 ± 4.4 y/36.2 ± 4.5 y)	ESHRE consensus criteria 2011 for poor responders	>45 y; FSH >20 IU/l; previous ovarian surgery; other causes of infertility (other than poor responder); male factor of infertility	GnRH long protocol; +treatment group 7.5 IU/d GH from day 21 of previous cycle until day of hCG triggering	Higher number of oocytes and embryos obtained, while no effect on implantation and pregnancy rates	11.8 ± 1.3	11.3 ± 1.2	**<0.001^a^**
Chen et al. (19)	No	Recurrent implantation failure (RIF) patients; Chinese	22/20 (33.9 ± 2.9 y/34.0 ± 3.4 y)	Normal hormone levels; no use of synthetic hormones >3 months prior to entry	Prior endometrial resection or endometrial polyps; antiphospholipid syndrome; infectious disease; hyperthyroidism; hyperprolactinemia; chromosomal abnormalities; thalassemia; male factors	GnRH; +treatment group 4 IU/d GH through stimulation until the day of hCG administration	Higher clinical pregnancy and live birth rates	11.6 ± 2.9	9.7 ± 1.5	**0.009^a^**
Liu et al. (24)	No	Normal responders; Chinese	781/781 (31.3 ± 3.6 y/31.3 ± 3.3 y)	Normal ovarian response; age 20–40 y; poor quality embryos in previous IVF/ICSI; repetitive fresh or frozen ET without pregnancy	Poor or high ovarian response; adjuvant therapy as DHEA, CoQ10; serious and unstable diseases (cardiovascular, cerebrovascular diseases); recurrent spontaneous abortion; male factor infertility	GH treatment group 2 IU/4 IU GH daily since day 2 of previous cycle (6 weeks GH pretreatment) or day 2 from ovarian stimulation until hCG trigger (2 weeks GH pretreatment)	Increased pregnancy rate	12.0 ± 2.2	11.6 ± 2.8	**0.036^a^**
**FROZEN EMBRYO TRANSFER/OOCYTE DONATION PROTOCOL**										
Wu et al. (43)	NA	Patients with thin endometrium; Chinese	32/30 (NA)	NA	NA	HRT; +treatment group subcutaneous injections of GH	Improved endometrial blood flow and increased endometrial thickness	8.8 ± 1.3	7.1 ± 1.9	**<0.05**
Yu et al. (54)	No	Patients with persistent thin endometrium; Chinese	5/5^*^ (32.2 ± 5.5 y) ^*^same women served as controls before entering GH treatment	Regular menstrual cycle; use of artificial cycle; endometrium ≥7 mm; no abnormalities with hysteroscopy; <40 y; pelvic tubal or male factor infertility	NA	HRT; +GH treatment with 4–5 intrauterine GH perfusions of 6 IU GH diluted with 0.5 ml 0.9% saline on 9th to 12th day of the cycle (bed rest 15 min)	Improved endometrial thickness and receptivity	8.0 ± 0.6	5.8 ± 0.7	**<0.05^b^**
Xue-Mei et al. (23)	No	Infertile women undergoing FET; Chinese	77 Gr1/ 77 Gr2/ 76 controls (cycles; *n* = 240 women) (30.3 ± 4.1 y/31.3 ± 5.0 y/30.7 ± 4.3 y)	≤ 38 y; vitrified embryos not older than 2 y; ≥2 embryos frozen	Congenital or acquired uterine malformation; endometrial polyps; submucosal fibroids; intrauterine adhesion; severe endometriosis or adenomyosis; diabetes mellitus; abnormal blood clotting	HRT with oral estradiol valerate from cycle day 3. +treatment group 1 (Gr1): 4 IU/d GH injections from cycle day 8 until prog injection; +treatment group 2 (Gr2): 4 IU/d GH injections from cycle day 3 until prog injection	Higher implantation, clinical pregnancy and live birth rates	9.2 ± 0.9 (Gr1); **9.6 ± 1.0 (Gr2)**	9.2 ± 0.8	**<0.001^b^**
Altmäe et al. (20)	Yes	RIF patients with fresh donated oocytes; Spanish	35/70 (42.2 ± 4.5 y/42.4 ± 3.7 y/43.8 ± 2.5 y) (35 GH RIF; Control Gr1 35 nonGH RIF; Control Gr2 35 pos controls undergoing 1st oocyte donation)	RIF (≥2 implantation failures); 30–51 y	NA	GnRH agonist + oral estradiol; +treatment group daily injections of 1 mg GH (~3 IU) for 10 days of proliferative phase induced by exogenous oral estradiol. 1–2 days later vaginal P treatment was started	Higher implantation, pregnancy and live birth rates	9.3 ± 1.5	8.6 ± 1.0 (Gr1 non-GH); 9.4 ± 1.7 (Gr2 pos control)	**0.046^b^**
Yang et al. (22)	No	Patients with thin endometrium; Chinese	184/61 (cycles; *n* = 225 women) (33.7 ± 3.6 y/33.7 ± 3.4 y)	<40 y; receiving 2 blastocysts; endometrial thickness <8 mm on prog administration day. All patients with hysteroscopy for adhesions before FET	Uterine malformations; severe endometriosis or adenomyosis; tumor; diabetes mellitus; immune abnormalities	GnRH agonist + estradiol valerate from day 2–3 of cycle+ vaginal estradiol after menstruation + prog for 5 days; + treatment group 4.5 IU GH every alternate day subcutaneously injected from day of prog administration until ET	Higher clinical pregnancy and implantation rates	6.6 ± 2.9	6.7 ± 0.7	0.24^c^
Cui et al. (21)	Yes	Patients with thin endometrium; Chinese	40/53 (29.8 ± 3.0 y/29.7 ± 3.6 y)	Endometrium ≤ 7 mm; <40 y; normal ovarian reserve; fresh ET canceled due to thin endometrium; ≥2 D3 embryos frozen	Uterine anomaly; intrauterine adhesion; endometrial polyp; adenomyosis; malignancy	Oral estradiol valerate from day 3 of cycle until day 18 + virginal estradiol on days 15–18 of cycle. +treatment group 5 IU/d GH subcutaneous injections cycle days 15–18	Higher implantation and clinical pregnancy rates	7.9 ± 0.7	6.3 ± 0.9	**<0.001^b^**

a *Day of hCG administration*.

b *Day of ET*.

c *Day of progesterone administration*.

*AMH, anti-Müllerian hormone; BMI, body mass index (kg/m^2^); COS, controlled ovarian stimulation; ET, embryo transfer; FET, frozen embryo transfer; GH, growth hormone; HRT, hormone replacement therapy; GnRH, gonadotropin-releasing hormone; hCG, human chorionic gonadotropin; hMG, human menopausal gonadotropin; ICSI, intracytoplasmic sperm injection; IU, international unit; NA, not applicable; PCOS, polycystic ovarian syndrome; Pos, positive; Prog, progesterone; RIF, recurrent implantation failure; RCT, randomized clinical trial. Bold text highlights the GH group of significant difference from control group*.

### Infertile Patients With Recurrent Implantation Failure

This is a group of patients that fail to achieve pregnancy in fresh or frozen embryo transfer cycles despite appropriate endometrial development (thickness and pattern) and good quality embryo transferred. Patients with recurrent implantation failure (RIF), having undergone three or more embryo transfer cycles after IVF treatment without a clinical pregnancy, are among the most difficult patients to treat, with no proven standard treatment. Impaired endometrial maturation is suggested as a common cause for RIF (56–58), making it a target patient group who could potentially benefit from GH co-administration during IVF procedure. Chen et al. study on 42 RIF patients undergoing IVF treatment found that GH treatment throughout the stimulation increased the endometrial thickness and consequent pregnancy and live birth rates among young patients <35 years old supplemented with GH when compared to no GH RIF group (19). Patients in both groups had similar peak estradiol levels and similar number of oocytes retrieved (19). While it is unclear if the difference in endometrial thickness of 11.61 ± 2.9 vs. 9.7 ± 1.46 mm between study and control groups, respectively was crucial in achieving higher pregnancy rates in the study group, the observation is nevertheless important. This has been reported again in the second study, a randomized clinical trial including 70 RIF patients in oocyte donation program (as an ideal model for assessing GH effect on patient's endometrium without confounding factors of ovarian age and response) (20). In that study patients, who were treated with GH throughout medicated frozen embryo transfer cycle demonstrated significantly thicker endometrium, 9.3 ± 1.5 mm vs. 8.6 ± 1.0 mm, respectively, and higher pregnancy and live birth rates compared with RIF patients in the placebo group (20) (Table 1).

These are the first two studies assessing GH effects on endometrium in RIF patients, and, although the findings are promising, clearly more studies on larger patient population, as well as randomized clinical trials (RCTs), are needed for any clinically meaningful conclusions. It is well-accepted that endometrial thickness does not necessarily mean that the endometrium is receptive, yet it is considered as a measure of endometrial maturity, and optimal growth of the endometrium (>7 mm) is required for a successful embryo implantation (59–61).

### Thin Endometrium

Infertile women with thin endometrium represent another potential patient population that could benefit from the GH administration. All studies on GH co-treatment during treatment of infertile women with thin endometrium were conducted in frozen embryo transfer (FET) cycles, where GH was administered during the endometrial preparation for FET (21, 22, 43, 54) (Table 1). The largest study by Yang et al. was conducted on 225 infertile women, and did not detect any significant GH effect on endometrial thickness, while reporting significantly higher clinical pregnancy and implantation rates (22). They assessed GH effect on endometrial thickness on the day of progesterone administration, which could explain the difference in their results from the rest of the studies. The other three studies all noted significant improvement in endometrial thickness on the day of embryo transfer among patients with thin endometrium after administering GH throughout the FET cycle (21, 43, 54), and significantly higher implantation and clinical pregnancy rates (21). Wu et al. study also detected improved endometrial blood flow in the GH-administered patient group (43), similar to later findings by Xue-Mei et al. study (23), who showed increased VEGF expression and improved perfusion of the uterine arteries in the group of infertile women treated with GH. In line with above, Cui et al. study detected VEGF up-regulation together with ITGB3 and IGF-1 in endometrial cells when exposed to GH (21). The state of high blood flow resistance and VEGF down-regulation with inadequate epithelial growth and vascularization have been described as pathophysiologic characteristics of thin endometrium (62), and subendometrial blood flow on the day of embryo transfer is related to the implantation and pregnancy rate in IVF (63). Cui et al. concluded that up-regulated VEGF in their study setting, in the GH group, partly resulted in the increase of subendometrial blood flow and thereby improved endometrial receptivity (21). Nevertheless, the exact mechanisms of GH actions on the endometrium and endometrial receptivity in general are to be unraveled in future studies. Also new studies with larger study groups and well-designed RCTs are required in order to clarify whether infertile women with thin endometrium benefit from the GH treatment.

### Poor Responders

Women with poor ovarian response in ART is another patient group where GH co-treatment in stimulation protocols have been studied. All these studies (see Table 1) have been RCTs, however with limited sample sizes, and all have reported beneficial effect of GH administration on the number and quality of oocytes and on the number of embryos obtained. Remarkably, while some improvement of endometrial thickness has been noted, those studies failed to show any beneficial effect on clinical pregnancy and live birth rates (13, 49–53). Based on these findings, one could conclude that GH co-treatment in poor responders with normal endometrium does not seem to have any significant impact on endometrial receptivity and hence pregnancy rates. Nevertheless, we should be cautious in drawing preliminary and potentially wrong conclusions in this type of studies without taking into careful consideration all potential confounders, including quality and number of embryos transferred, cleavage vs. blastocyst stage embryos and even type of luteal support provided in fresh and/or frozen embryo transfer cycles (64). In addition, the total productivity rate from a single oocyte retrieval is highest when more and better quality embryos are obtained, which can be exactly the case with GH-supplemented cycles in poor responders, resulting in higher cumulative pregnancy rates rather than per cycle success in this group of patients. Clearly, carefully designed large studies with transfers of single good quality embryo (fresh and frozen) are warranted, albeit quite challenging to perform, in order to clarify whether endometrial receptivity in infertile women with poor response in ART would benefit from GH administration.

### Normal Responders

Thus far, the largest group of infertile patients involved in studies on GH administration during IVF has been the normal responders (Table 1). The first study was performed on 240 infertile women undergoing FET, where two different GH supplementation protocols were compared—GH administration throughout the FET, and a single GH injection on day 8 of estrogen treatment (23). Notably, significant endometrial thickness improvement together with higher embryo implantation, clinical pregnancy, and live birth rates were detected among women with longer GH administration (23). The authors also noted that the longer GH addition to the treatment protocol increased the levels of estradiol, IGF-1, and VEGF serum levels, and improved perfusion of the uterine endometrial arcuate artery (23). The pulsatility index, resistance index, and peak systolic velocity/end diastolic velocity of the uterine arcuate arteries represent the resistance of blood flow from the point of measurement downstream; increased impedance of these arteries might correlate with poor endometrial receptivity and clinical outcomes (65).

The next studies analyzed 1,114 (16) and 1,562 (24) infertile women, respectively undergoing ovarian stimulation for IVF with GH co-administration throughout the stimulation, and a positive GH effect on endometrial thickness in addition to the higher clinical pregnancy rates was detected in study compared to control groups. GH effect on endometrial thickness was significantly increased among older infertile women of ≥35 years old compared to <35 years old, while both groups exhibited higher implantation and clinical pregnancy rates, most likely attributed to the higher number of high quality embryos obtained in GH-treated groups (16). In humans, changes in GH secretion could be age-related, as post-adolescence the secretion of GH decreases with age, which is why GH hyposecretion is observed in older patients (66). GH insufficiency can disrupt ovarian function and lead to reproductive difficulties (66). As mentioned above, in Du et al. study (16), GH-treated older women (≥35 years old) had implantation and clinical pregnancy rates more than two times higher than those observed during IVF cycles without GH. This result suggested that adding GH might be beneficial for older patients.

To conclude, research on the effects of GH co-treatment in ART among normal responders has been performed on sufficiently powered studies in terms of the sample size, nevertheless as all these studies were not randomized controlled trials, further well-designed research is needed to objectively assess the GH effect on ART outcomes in (young) women with normal ovarian reserve and normal response to ovarian stimulation.

### Future Perspectives

Further studies are warranted in order to determine the optimal dose, time, and duration of GH administration and to investigate the long-term safety of GH for patients and their offspring. The dosage and treatment duration of GH differed among conducted studies (see Table 1). Because of the limited experience with the GH co-treatment protocols, there is a lack of evidence to support the superiority of one over the other. In all the protocols used (see Table 1), GH was administered via subcutaneous injections, except for one study where GH intrauterine perfusion in 5 patients with non-responsive thin endometrium was successfully used (54).

Another crucial part is to define the appropriate patient population that would truly benefit from GH treatment for improving their uterine lining quality in terms of thickness and/or receptivity. GH seems to promote endometrial growth, and its use could be considered in women whose endometrium does not grow and/or mature sufficiently with standard treatment protocols. In addition, the current review concludes that even normal responders could potentially benefit from the GH administration in IVF programs, however, the improved pregnancy rates in some of the studies utilizing fresh IVF cycles could not be separated from improved embryo quality. While endometrial thickness and pattern upon GH administration has been recorded and reported, evaluation of endometrial receptivity is not as simple. Future studies need to focus on the molecular level in order to evaluate the endometrial transcriptome/proteome/secretome (67), with emphasis on receptivity markers to understand and clarify the possible mechanisms of GH on endometrial receptivity. An ideal setting would be to design an RCT with GH-supplemented mock cycles vs. control, during which endometrial receptivity could be studied on molecular level in detail (transcriptomics and/or use of commercially available endometrial receptivity tests; epigenomics and/or proteomics analyses). The mock cycle could be followed by a “true” FET cycle to enable evaluation and correlation to pregnancy rates. To sum up, undoubtedly more research on larger cohorts with carefully designed studies [as highlighted in a recent comment (64)] is needed to identify the patient group in whom the addition of GH to the treatment protocol in IVF programs will be most valuable.

Sample size and objectively designed studies (randomized clinical trials) is a delicate topic in ART as strict double-blind, placebo-controlled, RCTs are difficult to accomplish (68). It is extremely hard to perform fully blinded RCTs in IVF because of the patient recruitment issues, where aging women prefer not to participate in the placebo group that requires commitment for several months of their reproductive lifespan and which ultimately may not help them achieve pregnancy (68). Understandingly, patients tend to opt for any additional treatment, cost permitting, that would potentially help them to become pregnant. As a result, the studies of GH treatment effects on IVF outcomes are rather limited on its sample size and/or are retrospective or observational in nature; nonetheless, they provide important data concerning therapeutic interventions in IVF and open up future possibilities for improving infertility treatment protocols.

## Conclusions

The current review summarizes the recent data on GH co-treatment effects on endometrial parameters in assisted reproduction and proposes possible mechanisms of GH actions in the endometrium. Studies are indicating that co-treatment with GH could improve the endometrial thickness, and possibly receptivity among infertile women. This effect might occur through increasing endometrial blood perfusion and the expression of genes and proteins related to endometrial receptivity such as VEGF and ITGB3 together with IGF-1, however the exact mechanisms in the endometrium remain to be clarified.

Whether GH administration during IVF is useful and which patient groups could benefit from it needs further investigation, but the preliminary data suggest that women suffering RIF, patients with thin endometrium and older normo-responders could benefit from GH treatment when undergoing ART. Still, carefully designed and sufficiently powered cohort studies, RCTs, are required in the field in order to establish the most suitable therapeutic regimen for these patients and to clarify the confusion arisen from various studies that have shown either inconsistent or conflicting findings, used small patient cohorts and/or have been poorly designed with no blinding or placebo controls.

## Author Contributions

SA and LA equally contributed to the review idea and manuscript writing.

## Conflict of Interest

The authors declare that the research was conducted in the absence of any commercial or financial relationships that could be construed as a potential conflict of interest.
